# Microfluidic Mixing: A Review

**DOI:** 10.3390/ijms12053263

**Published:** 2011-05-18

**Authors:** Chia-Yen Lee, Chin-Lung Chang, Yao-Nan Wang, Lung-Ming Fu

**Affiliations:** 1 Department of Materials Engineering, National Pingtung University of Science and Technology, Pingtung 912, Taiwan; E-Mail: leecy@mail.npust.edu.tw; 2 Department of Vehicle Engineering, National Pingtung University of Science and Technology, Pingtung 912, Taiwan; E-Mails: clchang@mail.npust.edu.tw (C.-L.C.); yanwang@mail.npust.edu.tw (Y.-N.W.)

**Keywords:** active mixer, microfluidic mixing, passive micromixer

## Abstract

The aim of microfluidic mixing is to achieve a thorough and rapid mixing of multiple samples in microscale devices. In such devices, sample mixing is essentially achieved by enhancing the diffusion effect between the different species flows. Broadly speaking, microfluidic mixing schemes can be categorized as either “active”, where an external energy force is applied to perturb the sample species, or “passive”, where the contact area and contact time of the species samples are increased through specially-designed microchannel configurations. Many mixers have been proposed to facilitate this task over the past 10 years. Accordingly, this paper commences by providing a high level overview of the field of microfluidic mixing devices before describing some of the more significant proposals for active and passive mixers.

## 1. Introduction

Microfluidic devices have had a considerable impact on the fields of biomedical diagnostics and drug development, and are extensively applied in the food and chemical industries. The diminutive scale of the flow channels in microfluidic systems increases the surface to volume ratio, and is therefore advantageous for many applications. However, the specific Reynolds number (*Re =* 1 ρv/η) of liquid flows in such microchannels is very small. For example, the Reynolds number is of the order of 0.1 in a typical water-based microfluidic system with a channel width of 100 μm, a liquid flow rate of 1 mm/s, a fluid density of 1 g/cm^3^ and a viscosity of 0.001 Ns/m^2^. In such low Reynolds number regimes, turbulent mixing does not occur, and hence diffusive species mixing plays an important role but is an inherently slow process. Consequently, the aim of microfluidic mixing schemes is to enhance the mixing efficiency such that a thorough mixing performance can be achieved within shorter mixing channels, which can reduce the characteristic size of microfluidic devices. Furthermore, the development of efficient mixing schemes is essential for increasing the throughput of microfluidic systems and to realize the concept of micro-total-analysis systems and lab-on-a-chip systems.

Increasing the contact area between the species to be mixed is one of the most efficient means of enhancing the diffusive mixing effect. Accordingly, previous studies presented mixing schemes by feeding the samples of interest through discrete via holes, cantilever plate-valves or multi-channels in the microfluidic device. An alternative approach is to increase the contact area between the mixing species by designing the microchannel configurations so that the species are folded multiple times as they flow along the mixing channel. In passive mixing devices such as those described above, samples can typically be mixed within 55–300 ms, and hence the requirement for high device throughputs can be readily achieved. Besides increasing the contact area, the mixing performance can also be improved by increasing the time of contact between the multiple species. However, such schemes typically result in a lower mixing efficiency, and thus require a longer mixing channel to achieve a satisfactory mixing result (Table 1).

In contrast to the passive mixing schemes presented above in which the microchannel configuration is specifically designed to increase either the contact area or the contact time (or both) of the multiple species, active mixing schemes improve the mixing performance by applying external forces to the sample flows to accelerate the diffusion process [12]. Typically, active mixing schemes are implemented by incorporating some form of mechanical transducer within the microfluidic device using microfabrication techniques. For example, microfluidic mixers, which use embedded ultrasonic transducers to generate acoustic waves to stir the samples, have been shown to achieve a high mixing performance. However, acoustic vibrations also generate considerable heat that may lead to unwanted reactions between the samples. Various microfluidic devices with embedded microelectrodes for mixing the sample fluids dielectrophoretically have also been presented. The use of embedded electrode pairs has been shown to change the surface energy of the microchannel walls, providing an efficient means of enhancing species mixing by inducing local instabilities in the flow stream. The use of embedded electrode pairs has many of the advantages of active mixing schemes but does not require moving components, and hence results in cheaper and more reliable microfluidic devices (Table 2).

The microfluidic mixers presented above are all considered for the mixing of continuous bulk liquids. However, various discrete droplet-based mixing platforms have also been proposed. One such scheme involves the use of air pressure to form, actuate and mix two liquid droplets in a hydrophobic microcapillary valve device. Recently, many studies have presented mixing devices using liquid droplets based on electro-wetting phenomena. In these schemes, electro-wetting actuation is applied to separate liquid droplets from the bulk and to drive them to specific locations where they are repeatedly combined, mixed and separated. The microfluidic mixing of liquid droplets through the application of electro-wetting-induced droplet oscillations has also been demonstrated. These microfluidic mixing schemes take advantage of the open structure of the flow channel, allowing the mixed sample to be more easily transported to its required destination.

## 2. Active Microfluidic Mixers

**Figure 1.**
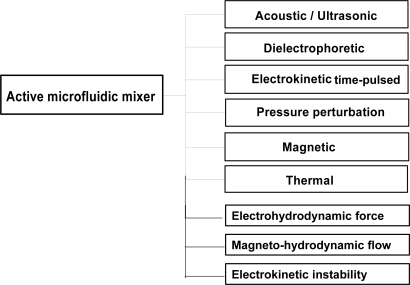
Categories of active microfluidic mixer.

Active microfluidic mixers enhance the mixing performance by stirring or agitating the fluid flow using some form of external energy supply. As shown in Figure 1, active mixers typically use acoustic/ultrasonic, dielectrophoretic, electrokinetic time-pulse, pressure perturbation, electro-hydrodynamic, magnetic or thermal techniques to enhance the mixing performance [1–11,26–57].

### 2.1. Acoustic/Ultrasonic Actuation [26–30]

Liu *et al.* [26] demonstrated the use of acoustically-induced microstreams to achieve mixing in a micro-chamber. In the design, a piezoelectric disk was used to excite air bubbles trapped in the top layer of the chamber at a frequency of 5 kHz. The mixing time was reported to be 6 s for a 40 V peak-to-peak excitation. Adopting a similar configuration, Yang *et al.* [27] employed a higher frequency of approximately 60 kHz and a 50 V peak-to-peak excitation in an attempt to accelerate the mixing process. However, the increased agitation of the fluid samples resulted in a temperature rise of 16 °C in the chamber. Tsao *et al.* [28] presented a Lamb wave micromixer in which Lamb waves were induced in the mixing species by a thin plate formed at one side of the mixing chamber and integrated with interdigitated transducers. Rife *et al.* [29] proposed a radio frequency-based microfluidic device with an operational frequency of 50 MHz that applied acoustic streaming to both pump and mix the sample species. Yaralioglu *et al.* [30] presented a micromixer featuring a simple microfluidic channel with embedded piezoelectric transducers and an operational frequency of 450 MHz. Through the application of an external electrical field, the transducers generated a strong acoustic streaming effect, enhancing species mixing within the channel.

### 2.2. Dielectrophoretic Force Actuation [31,32]

**Figure 3.**
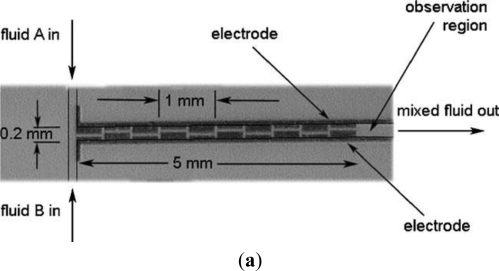

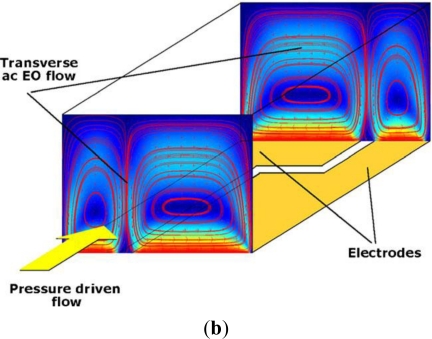
(**a**) Microphotograph of the DEP micromixer and (**b**) Schematic representation of circulating transverse flows (*rolls*) generated by AC electroosmosis which superimpose to the axial pressure driven flow within the micromixer. The positions of the asymmetric vortexes are twisted along the channel length in such a way as to implement a linked-twisted-map [3].

When the dielectrophoretic (DEP) force is applied to mixing, non-uniform alternating electrical fields induce the motion of polarized particles/cells [31]. The electrical field generates a dipole moment on the particles and the interaction between the induced dipole charges and the electrical field generates a net force which drives the particles either towards or away from the electrode. In a DEP micromixer, when the electrical field is combined appropriately in space and time with the velocity field, saddle point regions are generated within which sets of particles are stretched and folded about a virtual quasi-static point. The resulting chaotic motion leads to a rapid and efficient mixing effect. Campisi *et al.* [3] proposed a micromixer in which the liquids were electrokinetically displaced by generating rolls on co-planar electrodes through AC electroosmosis. A strong mixing performance was observed at 100 kHz and a 20 V peak-to-peak excitation (Figure 3).

### 2.3. Electrokinetic Time-Pulsed Actuation [4,5,33–39]

**Figure 4.**
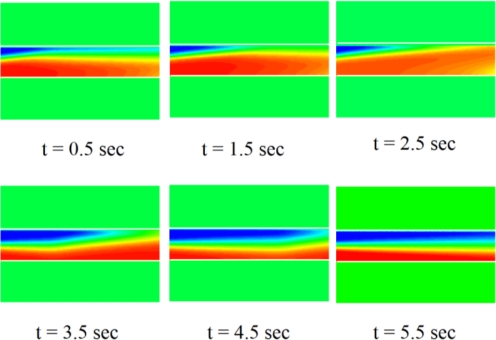
Sequence of concentration distribution in confluent stream mixing [33].

Electrokinetic time-pulsed microfluidic mixers apply an electrokinetic driving force to transport the sample fluids while simultaneously inducing periodic perturbations (Figure 4) in the flow field [33]. The performance of electrokinetic time-pulsed microfluidic mixers can be enhanced in a number of ways, such as by increasing the contact area and contact time of the sample streams, or by creating irregular flow fields in the mixing channel. Several microchannel configurations have been proposed for increasing the contact area in such devices, including T-shaped, cross-shaped, double-cross-shaped, and multi-T-shaped configurations. Electrokinetic time-pulsed microfluidic mixers typically apply either square or sine wave driving signals with frequencies varying from 0.1–5 Hz. Chen *et al.* [4] proposed a microfluidic mixing scheme in which the streams of species were mixed via the application of chaotic electric fields to the four electrodes mounted on the upper and lower surfaces of the mixing chamber. Mixing efficiencies of up to 95% were achieved in the micromixer.

### 2.4. Pressure Perturbation [40]

**Figure 5.**
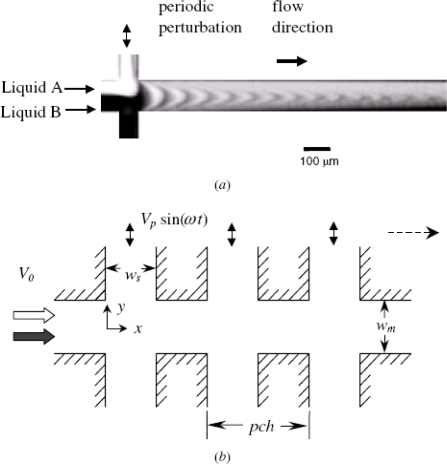
Model of a chaotic mixer with multiple side channels. (**a**) Experimental results of mixing in the device with one pair of side channels. The pressure perturbations induce lobe-like distortions of the interface and facilitate rapid mixing; (**b**) Schematic showing the new mixer with multiple side channels [40].

In pressure perturbation mixers, perturbations within the fluid streams are generated by velocity pulsing [40]. In a typical device, the mixer comprises a single main channel and multiple side channels, and the fluids within the main channel are stirred by velocity pulsing of the fluids flowing through the side channels. The resulting stretching and folding of the fluids in the main and side channels induce a chaotic advection effect, which enhances species mixing (Figure 5).

### 2.5. Electrohydrodynamic (EHD) Force [6,7,41]

In the micromixer presented by El Moctar *et al.* [41], two fluids with identical viscosity and density, but different electrical properties, were injected by syringe pumps, and the electrodes were arranged such that the electrical field was perpendicular to the interface between the fluid streams, creating a transversal secondary flow. The effects of both DC and AC electrical fields were investigated in a series of experimental trials. The results revealed that by applying an appropriate voltage and frequency to the electrodes, a satisfactory mixing performance could be achieved after less than 0.1 sec over a short mixing distance, even at a low Reynolds number of 0.02.

### 2.6. Thermal Actuation [8,42]

**Figure 6.**
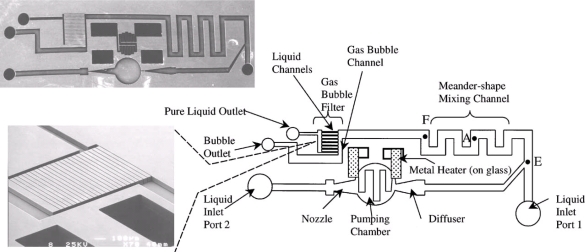
Schematic of the microfluidic system that includes a nozzle-diffuser-based bubble pump, a meander-shape fluid mixing channel and a gas bubble filter [42].

Tsai *et al.* [42] presented a microfluidic mixer incorporating a thermal bubble actuated nozzle-diffuser micropump, a meander-shape liquid mixing channel and a gas bubble filter (Figure 6).

The intrinsically oscillatory flow generated by the bubble actuated nozzle-diffuser micropump was shown to induce a wavy liquid interface, accelerating the mixing process. Xu *et al.* [8] showed the thermal mixing characteristics of two miscible fluids in a T-shaped microchannel .Thermal mixing processes in a T-shaped microchannel are divided into two zones, consisting of a T-junction and a mixing channel. The results show that the heat transfer process in the T-shaped microchannel can be divided into two heat transfer zones: The T-junction and the mixing channel. The flow rate ratio plays an important role in the thermal mixing process.

### 2.7. Magneto-Hydrodynamic Flow [43–45]

**Figure 7.**
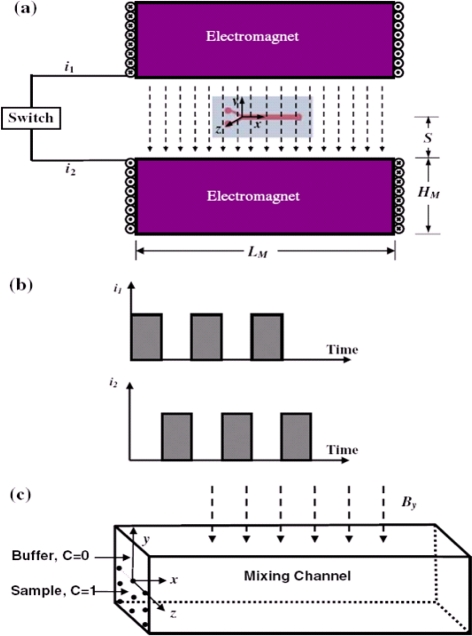
Schematic diagram of: (**a**) magnetic micromixer; (**b**) time-dependent current applied to the electromagnets and (**c**) mixing microchannel [9].

The magneto-hydrodynamic (MHD) flow effect has been used by various researchers to realize micromixers. For example, Bau *et al.* [43] developed an active micromixer that used either DC or AC electrical and magnetic fields to generate Lorentz forces. These forces induce MHD flows in an electrolyte solution and result in a stretching and folding of the fluid in the mixing chamber, mixing the species within several seconds. An elaborate micromixer based on MHD, reminiscent of the original arrangement of two blinking vortices used to demonstrate chaotic advection, was proposed by Yi *et al.* [44]. The device consisted of a small cylindrical chamber with an electrode deposited on its side wall and two copper-wire electrodes placed eccentrically inside the chamber on its lower surface. The chamber was positioned within a uniform magnetic field orientated parallel to the axis of the cylinder, and mixing was induced by alternately applying a potential difference for a period *T* between one of the wire electrodes and the circular side-wall electrode and then between the second wire electrode and the side-wall electrode. Particle tracing revealed that chaotic flows were induced and resulted in a satisfactory mixing result within 40 periods. Wang *et al.* [9] developed a magnetic particle driven micromixer in which mixing was induced by alternating the actuation of magnetic particles suspended in the fluid (Figure 7). The numerical results revealed that maximum efficiency was obtained at a relatively high operating frequency for large magnetic actuation forces and narrow microchannels.

### 2.8. Electrokinetic Instability [10,11,46–48]

**Figure 8.**
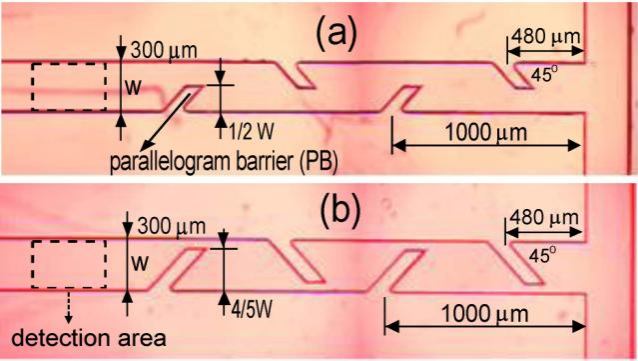
Microscopic images of microfludic mixer with parallelogram barriers in mixing channel [48].

The use of electrokinetic instability (EKI) as a mixing technique for electrokinetically-driven microfluidic flows with conductivity gradients has received considerable attention in recent years [46,47]. In an attempt to improve the mixing performance of micromixers, Tai *et al.* [48] developed a T-type mixer with parallelogram barriers formed within the microchannels (Figure 8). The authors presented experimental results for the sample concentration distribution in a micromixer for a parallelogram barrier length (PB) of 4/5 W (where W is the channel width), a 10:1 conductivity ratio, and electrical field intensities of 150 V/cm, 300 V/cm and 500 V/cm. The results indicated that the electrokinetic instability phenomenon was induced when high electrical field intensity was applied to drive the flow streams. When the electrokinetic instability effect was not induced, the mixing efficiency at a cross-section located 2.3 mm downstream of the T-junction was 60%. However, when the parallelogram barrier was established, the mixing efficiency increased to 91.25% at the same location. Recent years have witnessed many developments in active mixing approaches for microfluidic devices. Micromixer design appears to be moving towards active chaotic mixers with no moving parts. To avoid complicated microfabrication processes, and to reduce the cost and complexity involved in integrating active mixers in microfluidic systems, the species samples should be driven using an appropriate external energy source such as electrokinetic forces. In recent years, electrokinetic forces have been widely employed in many active mixers, such as the electrokinetic instability micromixer presented by Huang *et al.* in [11]. However, this particular design suffers the drawback of requiring a high voltage. Accordingly, low-voltage electrokinetic, AC electrokinetic and nonlinear electrokinetic techniques have received increasing attention in recent years as potential means of overcoming this limitation. The high flow rate or high velocity can also be produced through various nonlinear electrokinetic external sources. Therefore, the application of nonlinear electrokinetics to realize active mixers is likely to emerge as a major research topic in the microfluidics community in the future.

## 3. Passive Microfluidic Mixers

Passive micromixers contain no moving parts and require no energy input other than the pressure head used to drive the fluid flows at a constant rate. Due to the inherently laminar characteristics of micro-scaled flows, mixing in passive micromixers relies predominantly on chaotic advection effects realized by manipulating the laminar flow within the microchannels or by enhancing molecular diffusion by increasing the contact area and contact time between the different mixing species. Figure 9 summarizes the major forms of passive microfluidic mixing schemes [13–25,58–90].

### 3.1. Lamination [13,14,58–60]

**Figure 10.**
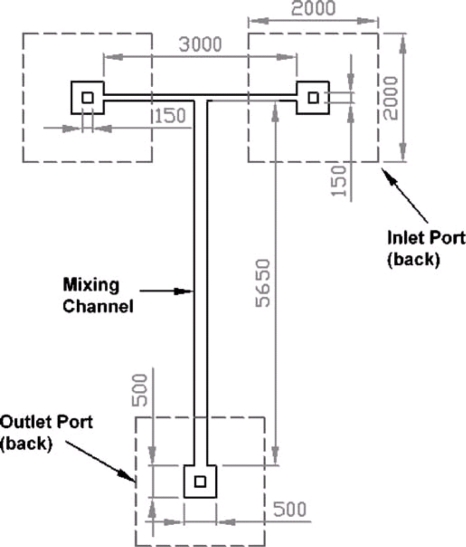
Schematic diagram of micro T-mixer [60].

**Figure 11.**
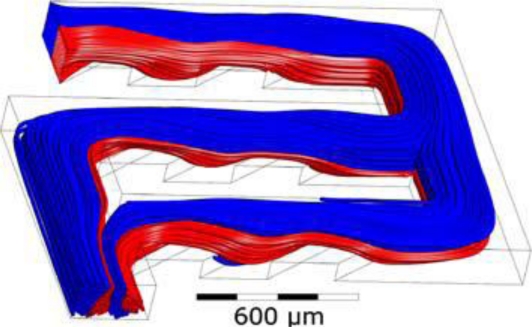
Simulated flow field in one mixing module showing lamination [14].

In miniaturized flow systems with Reynolds numbers varying from 2 to 100, flow structures can be artificially induced, assisting flow segmentation through inertia effects. In the zigzag channel considered in [58], segmentation was achieved through shear forces. The results showed that mixing was achieved within 1 s for fluid flows with a Reynolds number of 33 for two fluid streams flowing alongside each other in a microchannel of dimensions 300 μm × 600 μm × 100 mm containing 80 zigzags [58]. Another example of mixing via inertia forces was demonstrated by Scampavia *et al.* [59] who fabricated a coaxial mixer containing an inner core liquid stream with a low flow rate and an outer flow stream with a higher flow rate. The authors showed that the design achieved an acceptable mixing result within 55 ms for fluid flows with a Reynolds number of 5. Wong *et al.* [60] presented a micro T-mixer fabricated on a silicon substrate and bonded to a Pyrex glass plate (Figure 10). The experimental results revealed that a mixing channel with a hydraulic diameter of 67 μm and an applied pressure of 5.5 bar was sufficient to achieve complete species mixing in less than 1 ms after the initial contact between the two species flows with Reynolds numbers of 400–500. Buchegger *et al.* [13] proposed a horizontal multi-lamination micromixer based on wedge shaped inlet channels. The experimental results revealed highly uniform fluid mixing in the low millisecond second range. Tofteberg *et al.* [14] fabricated a passive micromixer that made a controlled 900 rotation of a flow cross-section followed by a split into several channels (Figure 11). The flow in each of these channels was rotated a further 90° before a recombination doubled the interfacial area between the two fluids, and the process was repeated the desired degree of mixing was achieved.

### 3.2. Intersecting Channels [61–63]

**Figure 12.**
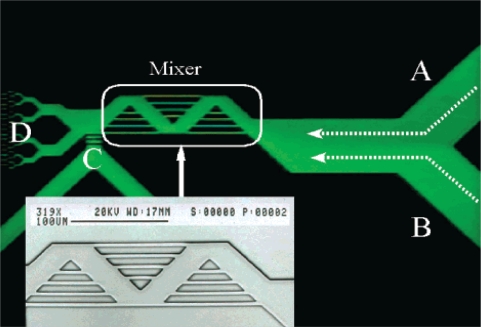
Photomicrograph of picoliter-volume mixer [62].

**Figure 13.**
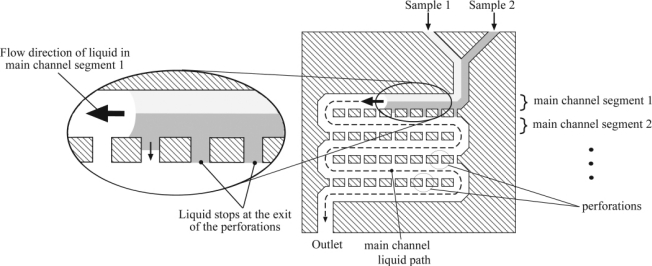
Top view of micromixer as two samples enter the first main channel segment [63].

Micromixers with intersecting channels can be used to split, rearrange and combine component streams to enhance mixing [61]. He *et al.* [62] proposed a picoliter-volume mixer with intersecting channels of various lengths and a bimodal width distribution (Figure 12). In the proposed design, all of the channels running parallel to the flow direction had a width of 5 μm, while those intersecting the parallel channel network had a width of 27 μm and were aligned at an angle of 450. The microchannels were designed so that the two species streams merged into one larger stream and then flowed together along a mixing channel of length 300 μm. The results showed that this configuration enabled complete species mixing within a distance of 200 μm, representing a considerable improvement compared to the mixing distance of 3000 μm required to fully mix the two streams in a conventional straight channel. Melin *et al.* [63] presented a micromixer in which a constantly changing, time-dependent flow pattern was created within a two sample liquid plug as the plug passed through a planar mixing chamber (Figure 13). The micromixer was shown to create a larger mass transfer within the liquid plug than that achieved in many of the passive devices presented previously. The proposed mixing chamber contained a main meandering channel with perforated walls that formed connecting channels between parallel segments of the main meandering channel. In the mixing process, two discrete liquid samples were fed into the mixing chamber laminarly. When the liquid wetted one side of the first perforated wall segment, it entered the perforation, but was prevented from exiting by surface tension effects. However, as the bulk of the liquid proceeded on to the next main channel segment, the other side of the perforated wall was wetted. As a result, the liquid/air interface at the exit of the perforation was replaced by a liquid/liquid interface, and hence the liquid flowed freely through the perforation. The same phenomenon was repeated in each of the main channel segments as the liquid flowed progressively through the mixing chamber. As a result, the constantly-changing flow lines within the liquid plug caused a self-folding effect within the plug, yielding a significant improvement in mixing performance.

### 3.3. Zigzag Channels [64,65]

**Figure 14.**
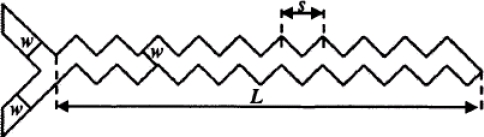
Dimensions of the microfluidic system integrating a “Y” junction with channel width *w*, linear length of the periodic step *s*, and linear length of the zigzag microchannel *L* [64].

Mengeaud *et al.* [64] presented a 100 μm wide zigzag microchannel integrating a “Y” inlet junction (Figure 14). The effect of the periodic step value *s* on the mixing efficiency was investigated in a series of experimental trials. The results indicated that for *Re* = 0.26, the mixing efficiency increased from 65% to 83.8% as the geometry ratio *s*/*w* was increased from 1 to 8. For low values of *s*/*w*, the number of angles increased, resulting in an increase in the effective width and a reduction in the effective length. For low Reynolds number flows, the most efficient zigzag configuration corresponding to *s* = 800 μm obtained a mixing efficiency of 83.8%. For *Re* = 267, the mixing efficiency increased rapidly to 98.6% as the geometry ratio was increased to 4, but reduced slightly to 88.1% as the geometry ratio was further increased, thus indicating the existence of an optimal zigzag geometry. In [65], Hong *et al.* presented a passive micromixer with a modified Tesla structure. In the proposed design, the species streams flowed close to the angled surface due to the Coanda effect, and this effect was used to guide the fluid streams to collide with one another. Mixing cells in opposite directions were then used to repeat the transverse dispersion caused by the flow impact. In the micromixer, one of the fluids was divided into two sub-streams and one of these two sub-streams was then merged with the second fluid stream from the main channel in the micromixer. The two streams were then mixed with the second sub-stream, resulting in a strong impact around the sub-channel of the micromixer. The results showed that the micromixer attained an excellent mixing performance at higher flow rates, and was characterized by a pressure drop of less than 10 kPa for flow rates of approximately 10 μL min^−1^. However, at lower flow rates, the mixer was constrained to the diffusive mixing regime, and hence the mixing performance was limited.

### 3.4. Three-Dimensional Serpentine Structures [16–18,66–70]

**Figure 15.**
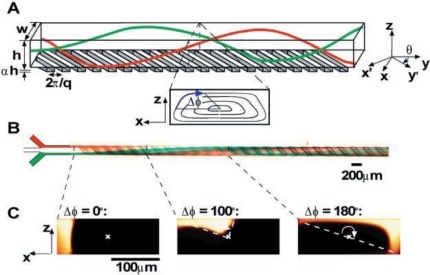
(**A**) Schematic diagram of channel with ridges; (**B**) Optical micrograph showing a top view of a red stream and a green stream flowing on either side of a clear stream in the channel and (**C**) Fluorescent confocal micrographs of vertical cross sections of the microchannel [66].

**Figure 16.**
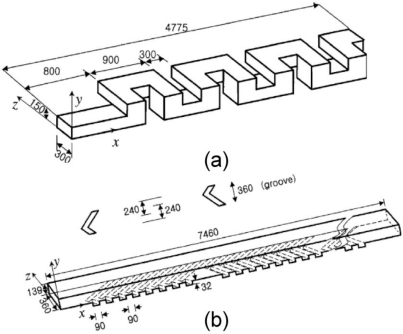
Geometry of: (**a**) three-dimensional serpentine and (**b**) stagger herringbone mixers [68].

**Figure 17.**
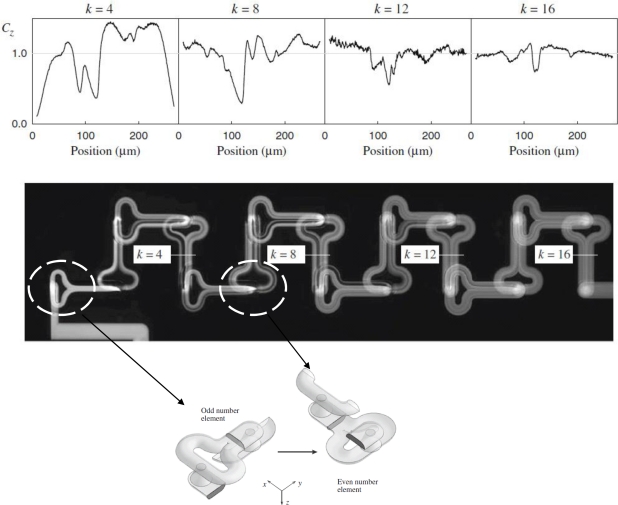
Composite microscope image of fluorescence intensity and profiles of measured concentration at selected element outlets (*k*) derived from the images [16].

Stroock *et al.* [66] proposed an active micromixer based on electrohydrodynamic (EDH) forces. In such mixers, an EDH force is created by applying an electrical field to a bulk flow in which both an electrical conductivity gradient and a permittivity gradient exist (Figure 15). Vijayendran *et al.* [67] presented a three-dimensional serpentine micromixer designed to induce a chaotic mixing effect. The mixing efficiency of the serpentine microchannel was observed to be twice that obtained in a conventional straight microchannel. Liu *et al.* [68] considered a three-dimensional serpentine mixer, and a staggered herringbone mixer (Figure 16), and performed numerical simulations to investigate the mixing characteristics of the two devices for different Reynolds number regimes and fluorescent sample mass fractions. At *Re* = 1, the mixing performance of both mixers varied inversely with the mass fraction of the sample due to the dominance of molecular diffusion. However, when the Reynolds number was increased to 10, the inverse trend was observed in the serpentine mixer. This phenomenon was attributed to an enhanced flow advection effect at large sample mass fractions. However, this effect was not observed in the herringbone mixer when the Reynolds number was increased over a similar range. Liu *et al.* [69] fabricated a three-dimensional serpentine micromixer featuring a “C-shaped” repeating unit designed to induce chaotic advection. The results showed that for flows with a Reynolds number of 70, the mixing efficiency in the serpentine channel was 16 times higher than in a conventional straight channel and 1.6 times better than in a zigzag channel. Chen *et al.* [16] investigated a folding flow micromixer in the Stokes flow regime (Figure 17). Both the simulated and experimental results revealed a significant effect on mixing from a small misalignment of the glass layers that formed the mixer geometry. A layer offset of 5 μm (1.5% of channel width) produced a variation of up to a 26% in the measurement of the mixture uniformity, and improved or worsened depending on the precise offsets of the layers. In 2009, Kamg *et al.* [17] simulated and optimized a set of variables (*i.e*., sense of rotation of two rotational flows, aspect ratio of channel and ratio of bypass channel to whole width) and found at proper combination of the variables, almost global chaotic mixing was observed in the Stoke flow regime. Moon *et al.* [18] presented a strategy for the forced assembly of immiscible polymer into targeted structures via development of a planar polymer micro-mixer. The mixer drove streams of molten polymer through mixing chambers, which were fabricated from metal shims that contained flow channels. By stacking the shims, complex 3D mixing flows could be generated. The advantages of the mixing technology include sample sizes significantly less than traditional micro-mixers (<100 mg), simple reconfiguration of the flow geometry and optical access to the flow.

### 3.5. Embedded Barriers [21,71–73]

**Figure 18.**
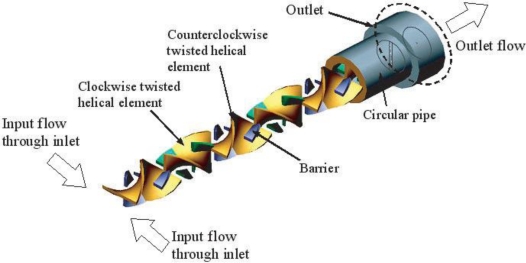
Schematic diagrams of barrier embedded Kenics micromixer [72].

Keoschkerjan *et al.* [71] fabricated a micro-reaction unit for chemical engineering applications based on the combination of multi-lamination and chaotic advection effects. In the proposed design, a mixing zone with a three-dimensional geometry was formed through overlapping microrestrictions. The mixing zone of the second micromixer was formed through cavities in the two wafer levels. The cavities were arranged to form a continuous three-dimensional channel. The geometry of the three-dimensional channel forced the fluid to follow a tortuous path and induced a permanent change in the flow direction. The mixing performance was further enhanced by the turbulence-like and restriction effects induced at the corners of the three-dimensional channel. Kim *et al.* [72,73] developed barrier embedded micromixers for pressure-driven flow in which chaotic flow was induced by applying periodic perturbations to the velocity field via periodically inserted barriers along the upper surface and helical type flow structures were induced by slanted grooves on the lower surface of the microchannel (Figure 18). Observations using a confocal microscope revealed cross-sectional mixing behaviors in several locations in the micromixer. The proposed design was validated experimentally at a flow rate corresponding to a Reynolds number of 2.28 (corresponding to *Re* = 1.24 × 10^4^ with a Rhodamine diffusivity of 2.8 × 10^−10^ m^2^s^−1^). Laser scanning of the entrance zone of the micromixer identified a bright image only in the half-zone containing Rhodamine. Bright images were also observed at the no-barrier zone in the first half-cycle, thus confirming the cross-sectional rotating flow effect induced by the slanted grooves. When the streams entered the barrier zone in the next half-cycle, laser scanning showed that the flow had rotated yet further. The experimental results confirmed that the barrier embedded micromixer yielded excellent species mixing within a short length of channel. Recently, Singh *et al.* [21] analyzed and optimized different designs of SMX motionless mixers based on the Mapping Method. Three design parameters that constituted the number of cross-bars over the width of channel, *N_x_*, the number of parallel cross-bars per element, *N_p_*, and the angle between opposite cross-bars. An optimum series for all possible SMX(*n*) designs to obey the universal design rule is *N_p_* = (2/3) *N_x_* − 1, for *N_x_* = 3, 6, 9, 12…

### 3.6. Slanted Wells [74–76]

**Figure 19.**
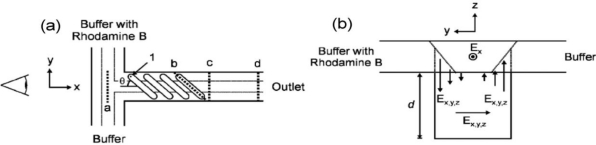
(**a**) Top-view schematic of the simulation geometry for T-channel; (**b**) End-view schematic of the simulation geometry as viewed down the line of sight of (a) along with arrows depicting the general direction of the electric field within the slanted wells. *Ex*, *Ey*, and *Ez* are the components of the electric field and *d* is the depth of the channel [75].

Johnson *et al.* [74,75] presented a micromixer incorporating slanted wells at the inlet junction. The presence of these wells led to a high degree of lateral transport within the channel and ensured a rapid mixing of two confluent streams undergoing electroosmotic flow (Figure 19). The micromixer was shown to successfully mix streams with a low flow rate (0.06 cm/s, ≥75% mixing), but had varying degrees of success mixing streams traveling at a higher flow rate (0.81 cm/s, 45–80% mixing). Yang *et al.* [76] investigated the effects of the asymmetry index *p* and depth ratio *α* of the groove on the mixing performance of a staggered herringbone mixer with patterned grooves. Because the two vortices within the mixing channel are determined by the asymmetry index *p*, vortices with dissimilar scales were shown to provide a better mixing performance than two equally-sized vortices. Furthermore, the results showed that the intensity of the vertical fluid motions at the side edges of the grooves increased with increasing groove depth *α*, resulting in a significant improvement in the mixing effect.

### 3.7. Twisted Channels [23,77–79]

**Figure 20.**
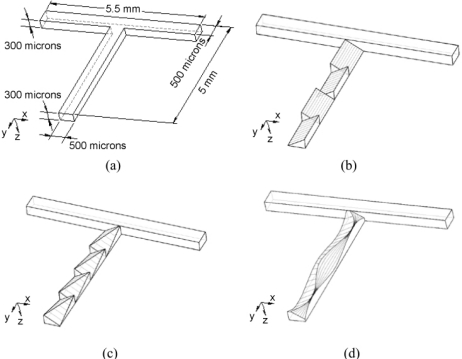
(**a**) Schematic diagrams (upside down) of (a) T-mixer; (**b**) inclined mixer; (**c**) oblique mixer and (**d**) wavelike mixer [77].

Jen *et al.* [77] presented a micromixer featuring a three-dimensional structure of twisted microchannels designed to induce a chaotic mixing effect within the fluid streams. In addition to the conventional T-mixer configuration, the authors also investigated three other types of micromixer featuring inclined, oblique and wavelike microchannels (Figure 20). The twisted microchannels were designed such that the angle of the channels’ lower surfaces alternated in each longitudinal subsection of the mixing channel. Hence, the fluid flow sways from side to side as it travels along the microchannel, resulting in a chaotic advection effect. Park *et al.* [78] presented a breakup method based on passive rotation for enhancing the mixing efficiency of micromixers. The proposed method not only actively generated interfaces between the mixing species, but also enhanced the diffusion process at the interface. The micromixer actually incorporated two separate mixing regions, one region with shorter segments of length 200 μm and a second region with longer segments of length 400 μm. The results showed that this design caused a rotation of the fluids flowing through the channel. The fluids were observed to mix well and the interface was distorted at high Reynolds numbers. Conversely, at low Reynolds numbers, the fluids were hardly mixed and the interface was not distorted; only the respective positions of the two fluid streams were changed. Hardt *et al.* [23] presented a low-Reynolds number split-and-recombine mixer. In their design, when fluid 1(2) entered the upper (lower) half of the stream, it was directed into the left (right) branch. The two streams were then recombined and directed into a channel with a cross-section identical to that of the inlet of the mixing element. In this way, the number of fluid lamellae doubled after each step and the average lamella thickness halved. In 2005, Aubin *et al.* [79] presented staggered herringbone groove micromixers that had an off-centered herringbone pattern on the lower surface of the microchannel, creating a transverse velocity component in the flow field. Such mixers comprised several mixing cycles, where each cycle comprised two sequential regions of grooves, (two half-cycles). The asymmetry direction of the herringbone pattern switched with respect to the centerline of the microchannel every half cycle. For improved mixing efficiency, the depth of the grooves in staggered herringbone micromixers was specified at 30% of the channel height. This promoted both spatial homogenization and a reduction of the striation thickness without increasing the pressure drop across the mixer.

### 3.8. Surface-Chemistry Technology in Microchannels [80–83]

**Figure 21.**
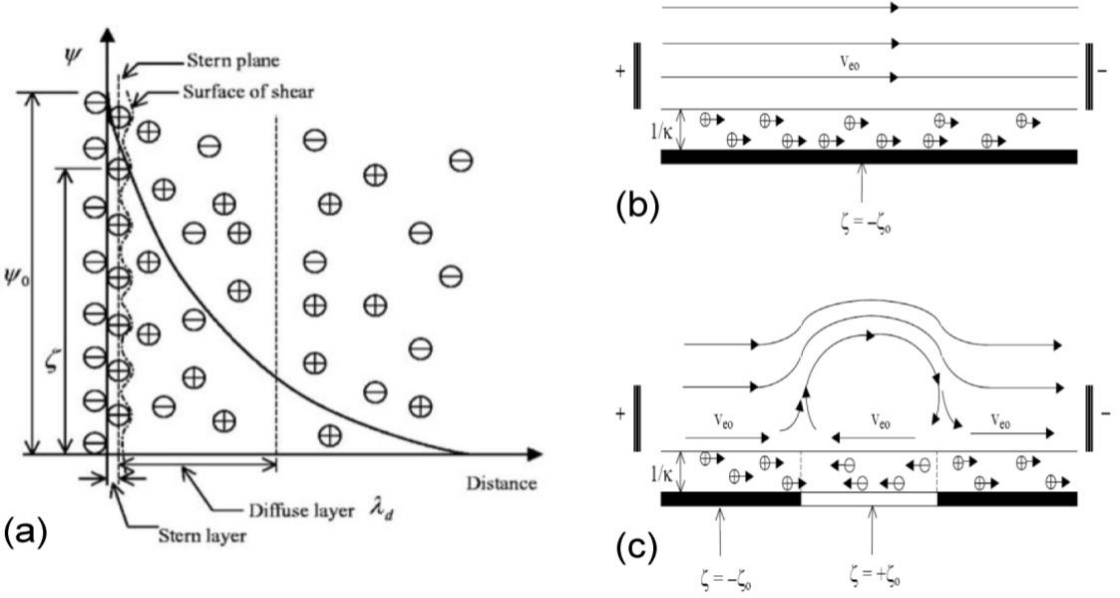
Schematic illustration of electrical double layer (EDL) and electroosmotic flow near the EDL: (**a**) the EDL next to a negatively charged solid surface (*ψ* is the EDL potential, *ψ*_0_ is the surface electric potential, *ζ* is the zeta potential); (**b**) a homogeneous surface (*ζ* = −*ζ*_0_) and (**c**) a homogeneous surface with a heterogeneous region (*ζ* = +*ζ*_0_), *ζ*_0_ *>* 0 [82].

In microfluidic systems, very high pressure gradients are generally required to drive and manipulate the fluid flow. Due to the small characteristic scale of the microchannels in typical microfluidic devices, surface forces dominate, and high friction effects are generated. Conventionally, microchannels are fabricated using silicon dioxide. Silicon dioxide surfaces are typically negatively charged due to their deprotonated silanol groups (≡Si–O^−^). When these surfaces come into contact with a solution containing ions, the positive ions are attracted to the surface, forming an important diffuse layer [80]. If an electrical field is then applied, the diffuse layer of positive ions moves in the direction of the applied field. The movement of the diffuse layer drags the bulk fluid into motion via momentum coupling, resulting in the so-called electro-osmotic flow (EOF) phenomenon [81]. Mixing in electroosmotic flow is generally diffusion-dominated. However, the introduction of electrically charged surface heterogeneities enhances the mixing efficiency by creating localized flow circulation regions (Figure 21) [82]. By selectively patterning positively charged molecules on a negatively charged channel wall, flow vortices can be induced from the differences in electrostatic potential between the homogeneous and heterogeneous surfaces. These localized flow vortices can be exploited to yield a significant improvement in mixing performance over a variety of microchannel configurations, including in-line, staggered, serpentine, herringbone and diagonal arrays [83]. In numerical investigations, the authors specified the patch length and spacing parameters required to maintain a constant ratio of heterogeneous-to-homogeneous surface areas over a channel length of 1.8 mm. The results revealed that the nonsymmetrical heterogeneous patterns, namely the staggered and diagonal patterns, generated a higher mixing performance than either the symmetrical herringbone pattern or the in-line arrangement. With a theoretical mixing efficiency of 96%, the staggered configuration provided the highest mixing performance, outperforming the diagonal, herringbone and serpentine configurations by 8%, 31% and 36%, respectively. Furthermore, compared to the homogeneous case, the staggered configuration provided a 61% improvement in mixing efficiency. The use of heterogeneous surface charge patches to manipulate electrokinetic flows provides a simple, low-cost solution to mixing problems in lab-on-a-chip systems, and is therefore likely to receive increasing attention in future studies.

## 4. Conclusions

Advances in MEMS techniques in recent decades have enabled the fabrication of sophisticated biochips for a diverse range of applications. Compared to their traditional macro-scale counterparts, micromixers have a shorter operation time, a lower cost, an improved portability and a more straightforward integration with other planar bio-medical chips. This paper has presented a systematic review of the major micro-mixers presented in the literature over the past 20 years or so. It has been shown that depending on their mode of operation, these micromixers can be broadly categorized as either active or passive. The operational principles and mixing performance of each type of micromixer have been systematically discussed, and their relative advantages and disadvantages highlighted where appropriate. Overall, the results presented in this review confirm the applicability of micromixers for a diverse range of low-cost, high-performance microfluidic applications.

## Figures and Tables

**Figure 2.**
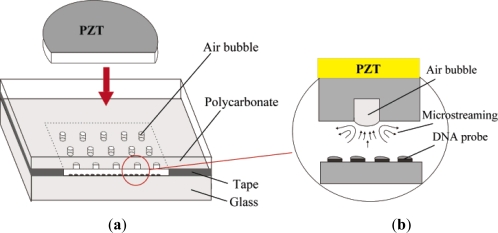
Schematic showing a number of air pockets in the top layer of the DNA biochip chamber: (**a**) overview; and (**b**) side view [26].

**Figure 9.**
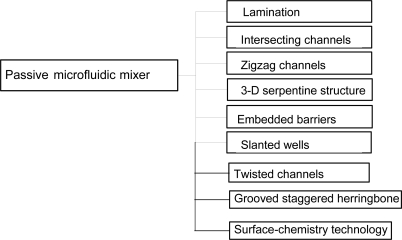
Categories of passive microfluidic mixer.

**Table 1.** Performance of active micromixers in recent five years [1–11].

**Categories**	**Mixing Technique**	**Mixing Time (ms)**	**Mixing Length (μm)**	**Mixing Index**	**Reference**
Acoustic/Ultrasonic	Acoustically driven sidewall-trapped microbubbles	120	650	0.025	[1]
	Acoustic streaming induced by surface acoustic wave	600	10,000	0.9	[2]
Dielectrophoretic	Chaotic advection based on Linked Twisted Map	-	1000	0.85	[3]
Electrokinetic time-pulsed	Chaotic electric fields	100	Width * 5.0	0.95	[4]
	Periodic electro-osmotic flow	-	200	0.88	[5]
Electrohydrodynamic force	Staggered herringbone structure	-	825	0.2	[6]
	Staggered herringbone structure	-	2300	0.5	[7]
Thermal actuation	Thermal	-	6000	-	[8]
Magneto-hydrodynamic flow	High operating frequency	1100	500	0.977	[9]
Electrokinetic instability	Low Reynolds number	-	1200	0.98	[10]
	Low Reynolds number	-	1200	0..98	[11]

**Table 2.** Performance of passive micromixers in recent five years [13–25].

**Categories**	**Mixing Technique**	**Mixing Time (ms)**	**Mixing Length (μm)**	**Mixing Index**	**Reference**
Lamination	Wedged shaped inlets	1	1	0.9	[13]
	90° rotation	-	-	0.95	[14]
Zigzag channels	Elliptic-shape barriers	-	10,000	0.96	[15]
3-D serpentine structure	Folding structure	489	-	0.01	[16]
	Creeping structure	-	-	0.015	[17]
	Stacked shim structure	-	-	-	[18]
	Multiple splitting, stretching and recombining flows	-	-	-	[19]
	Unbalanced driving force	-	815ψ	0.91	[20]
Embedded barriers	SMX	-	-	-	[21]
	Multidirectional vortices	-	4255	0.72	[22]
Twisted channels	Split-and-recombine	730	96,000	∼1	[23]
Surface-chemistry	Obstacle shape	-	1000	0.98	[24]
	T-/Y- mixer	-	1000	0.95	[25]
